# Resetting the Narrative in Australian Aboriginal and Torres Strait Islander Nutrition Research

**DOI:** 10.1093/cdn/nzaa080

**Published:** 2020-05-18

**Authors:** Annabelle Wilson, Roland Wilson, Robyn Delbridge, Emma Tonkin, Claire Palermo, John Coveney, Colleen Hayes, Tamara Mackean

**Affiliations:** 1 College of Medicine and Public Health, Flinders University, Adelaide, South Australia, Australia; 2 Department of Health Professions, Swinburne University, Melbourne, Victoria, Australia; 3 Department of Nutrition, Dietetics and Food, Monash University, Melbourne, Victoria, Australia; 4 College of Nursing and Health Sciences, Flinders University, Adelaide, South Australia, Australia

**Keywords:** Aboriginal and Torres Strait Islander, Indigenous, nutrition, food, health, knowledges, colonization

## Abstract

As the oldest continuous living civilizations in the world, Aboriginal and Torres Strait Islander peoples have strength, tenacity, and resilience. Initial colonization of the landscape included violent dispossession and removal of people from Country to expand European land tenure and production systems, loss of knowledge holders through frontier violence, and formal government policies of segregation and assimilation designed to destroy ontological relationships with Country and kin. The ongoing manifestations of colonialism continue to affect food systems and food knowledges of Aboriginal peoples, and have led to severe health inequities and disproportionate rates of nutrition-related health conditions. There is an urgent need to collaborate with Aboriginal and Torres Strait Islander peoples to address nutrition and its underlying determinants in a way that integrates Aboriginal and Torres Strait Islander peoples’ understandings of food and food systems, health, healing, and well-being. We use the existing literature to discuss current ways that Australian Aboriginal and Torres Strait Islander peoples are portrayed in the literature in relation to nutrition, identify knowledge gaps that require further research, and propose a new way forward.

## Introduction

Aboriginal peoples have a profound connection to food and food practices as part of a sovereign and intimate relationship with Country. Past and ongoing colonization of Australia has affected this connection with clearing of traditional lands, introduction of foreign species, and forced introduction of ration-based diets (1). This was preceded by a forced removal and relocation of Aboriginal peoples from land including massacres and clearing of traditional lands for large-scale westernized agricultural production. Despite this, many Aboriginal peoples today hold knowledge about traditional food practices and include this as part of their lives, individually and collectively, demonstrating a desire to continue to apply knowledges that are of benefit to individuals and communities in sourcing foods that supplement Western-based diets, and demonstrating enduring strength and resilience. In Australia, Aboriginal and Torres Strait Islander peoples experience nutrition-related health conditions at much higher rates than the non-Indigenous population, for example rates of diabetes and kidney disease that are 3.3 and 3.7 times higher, respectively (2). We use a narrative synthesis of the literature to discuss 3 ways in which Australian Aboriginal and Torres Strait Islander peoples are currently portrayed in the peer-reviewed literature. The purpose of the narrative analysis was to compare and contrast existing research findings, to suggest new questions arising from the literature, and to provide a detailed picture of the Aboriginal and Torres Strait Islander nutrition area (3). Literature was included in the narrative synthesis if it discussed nutrition issues in relation to Aboriginal and Torres Strait Islander peoples in Australia. Through this synthesis, we argue that a shift in this narrative is required in order to redress fundamental incongruencies in evidence and best practice and consequent inequities in nutrition-related health conditions.

## Current Portrayal of Aboriginal and Torres Strait Islander Peoples in the Literature

### Framing of Aboriginal and Torres Strait Islander peoples’ nutritional heritage

Before colonization, Aboriginal peoples lived an ecological lifestyle, which involved high levels of physical activity and sophisticated agricultural (land management, plant farming, and animal husbandry) and aquacultural practices (1, 4). Diets were often seasonal, reflecting seasonal variability in the landscape, low in energy density, and high in nutrients. They were typically high in protein, complex carbohydrates, micronutrients, and polyunsaturated fats while low in sugars and saturated fat (5–7). There is little evidence suggesting that major health issues such as diabetes or cardiovascular disease were prevalent before colonization while maintaining traditional diets (8, 9). Although food sources were variable across space and time, necessitating movement through the landscape, evidence suggests that the nutritional profile remained stable (8). Food was distributed according to Traditional Law and based on cultural practices and kin relationships, with Elders and older people prioritized, and food distributed according to seniority and need (5). Food represented more than nutrition, with collection of food being an important social activity, an opportunity to pass down knowledge from Elders to younger people, and a vital way to care for and connect with Country (10).

Until recent times, it has been rare to encounter work that references the remarkable engineering feats of Aboriginal peoples used to intensify and refine agricultural and aquacultural techniques: the sophisticated paperbark and bamboo fish traps of the Glyde River; the Brewarrina Fish Traps, also being the oldest man-made structure on the planet (1); complex and integrated irrigation systems designed to irrigate grain fields on the Nicholson River were observed by Tindale in 1977; and in New South Wales construction of large dams by the Wiradjuri People was common, with dams deliberately stocked with crayfish and yabbies (1).

Despite this, Aboriginal and Torres Strait Islander peoples’ lifestyles before colonization are often framed in the peer-reviewed literature as “hunter gatherer” (11), grounded in a classical linkage of agriculture and property rights by theorists such as Adam Smith (among others) as markers of complex social development (12). By the late 18th century the linkage with agriculture was well established, and many believed “society without agriculture, was a society without property rights in land” (12, p. 102). When sailing the East Coast of the continent James Cook made a note that “the Natives knew nothing of Cultivation” [*sic*]; they were hunter gatherers, unlike the “farmers” of North America and Polynesia he had previously encountered (12, pp. 100–1).

This deliberately erroneous colonial narrative fails to recognize the complexities and intricacies of the food systems that were part of Aboriginal and Torres Strait Islander peoples’ lives for tens of thousands of years and reduces these complex engineered, intricate, nutritional and cultural food systems to a simplified nature grounded in a colonized discourse. Reductive discourses that simplify food systems, grounded within a colonial framework, are of no value when addressing nutrition-related health inequities for Aboriginal and Torres Strait Islander peoples. This colonized discourse provided for forms of genocide and dispossession and continues to do so across the landscape and all aspects of the lives of Aboriginal and Torres Strait Islander peoples, in varying forms, to this day. In the context of nutrition, although there is currently general will to directly address issues of Aboriginal and Torres Strait Islander health and nutrition, there continues to be a lack of recognition of these historical atrocities and the overly simplistic framing of precolonization Aboriginal and Torres Strait Islander food systems remains and is perpetuated in the current nutrition literature.

### Lack of recognition of nutritional colonization

As a part of colonization, access to traditional foods was denied and Aboriginal peoples were forced to eat a diet higher in refined carbohydrates and saturated fats (11, 13). In appalling acts of genocidal violence and as a form of social control, food and water were used as a vehicle to poison Aboriginal peoples. In the mid-nineteenth century, a method of distributing rations 3 times/d was widely used across the continent (13, 14). These rations consisted of flour, rice, sugar, and tinned or salted meats, with fruit, vegetables, and fresh meat/seafood provided occasionally (13). The mission and town camp systems acted as sites of control over the lives of Aboriginal peoples and the use of a ration system within this structure formed a part of a complex web of colonizing power, with food rations forming a central component of that power dynamic. The ration system negatively affected the health of Aboriginal peoples. The ration system became a system of control whereby the food supply could be controlled and manipulated, using hunger and starvation as tools to ensure Aboriginal peoples’ reliance on and compliance to the state. It undermined the ecological lifestyle and interrupted traditional social, cultural, and relational roles connected to food (13). Deliberate undermining and attempted interruption of knowledge transmission fostered a narrow conception of the Australian food system as a whole, and replaced it with a settler narrative of taming a harsh frontier wilderness, excluding the abundant ethnographic evidence of sustainable practices of farming and aquaculture and landscape management of Aboriginal and Torres Strait Islander peoples from this narrative.

Previous research has demonstrated the importance of an awareness of colonization in the practice of dietitians and other health professionals (15) and current policy regarding Aboriginal and Torres Strait Islander health also acknowledges this as a critical determinant of well-being. Today, many Aboriginal peoples associate food, in general, with traumatic colonization practices that are centered around the control of food and food systems which are then passed between generations (14). This cannot be ignored, and further research is required to explore and document what nutritional colonization looks like, and how it affects Aboriginal and Torres Strait Islander peoples’ engagement with food and the food system today.

### A deficit approach

Regularly, Aboriginal peoples are reported as having “poor diets” (11, 16) and the focus of much research and intervention reported in the peer-reviewed literature has been on what needs to be done to “fix” the “poor dietary intake” of Aboriginal peoples (11, 17). For example, the introduction of the cashless welfare card (18), which seeks to control the way in which predominantly Aboriginal communities spend money to ensure purchase of healthy food. This represents a deficit approach and further reinforces the narrative of Aboriginal peoples having poor diets based on poor personal choices and therefore needing government intervention. This stems from a deficit-based approach where Aboriginal peoples are seen to lack agency in determining their own nutritional needs and aspirations. A viable alternative to the cashless welfare card was identified in a recent review of Aboriginal and Torres Strait Islander nutrition (11) which highlighted the importance of community involvement and local context in developing nutritional interventions, the lack of a policy framework, and the need for a social determinants perspective (including issues of food security). However, this review also reinforced deficit-based and incorrect approaches including the notion of the “hunter gatherer,” “feast,” and “famine” and lacked insight into how Aboriginal peoples can be involved if their very existence and worldviews are excluded, marginalized, diminished, and antiquated (11).

It is important to acknowledge that the systems that fund research, and therefore much of the driver of what is researched, are inherently “problem focused” and therefore require deficits to be highlighted in order to justify the work. This is also the case in policy and publication models that emphasize the definition of problems rather than questions. We argue that there is a need to shift this narrative at all levels with research, policy, and practice, to ensure that the type of narrative that is valued is consistent and can therefore be enacted consistently by all.

## Shifting the Narrative

It is necessary to reset the portrayal of Aboriginal and Torres Strait Islander nutrition away from narratives which inaccurately frame the nutritional history of Aboriginal and Torres Strait Islander peoples, do not explicitly recognize the relation of past and ongoing colonization to food and nutrition, and have a deficit approach. It is established that a lack of inclusion of Aboriginal and Torres Strait Islander perspectives and knowledges in societal processes and practices affects the well-being of Aboriginal and Torres Strait Islander peoples (19). Therefore, shifting the narrative toward one which *1*) values, includes, and incorporates Aboriginal and Torres Strait Islander knowledges and understandings of food, nutrition, and health; *2*) is decolonizing; and *3*) is underpinned by a strengths-based approach, will enable proper understandings of and solutions to address the multifarious phenomena affecting the nutrition of Aboriginal and Torres Strait Islander peoples through partnering with Aboriginal peoples and integrating Aboriginal and Torres Strait Islander peoples’ understandings of food and food systems into the current narrative.

### A strengths-based approach

There has been a clear call from Aboriginal researchers that a strengths-based approach to research and health (including nutrition) is necessary. Instead of focusing on Aboriginal and Torres Strait Islander peoples as disadvantaged and problematic, it is necessary to focus on what is possible through processes of self-determination and cocreation of knowledge and shared experience regarding health and well-being (20–23). There has also been previous acknowledgement that although utilized in some fields of health, strengths-based approaches are underutilized in nutrition (21) and do need to be used (21, 24): “redressing the current imbalance between strengths and deficit-based approaches is needed” (21). It is also acknowledged that strengths- and deficit-based approaches can coexist and can both contribute important information, for example, both approaches are required to pragmatically address the *structural* causes of inequities (21). What is important is that the current approach gives only partial answers to complex phenomena and can reproduce and reinforce unhelpful colonial concepts, therefore innovation and rebalancing are required. However, where deficit discourse runs counter to the strengths-based narrative of Aboriginal food systems, knowledges, and the production of new ways of engaging with such knowledge systems, we propose that a deficit approach could be eliminated in an era where public health/nutrition research has greatly shifted toward a co-design process of coequal engagement, knowledge sharing, and production between and with Aboriginal and research communities.

### Valuing, including, and incorporating Aboriginal and Torres Strait Islander knowledges and understandings of food, nutrition, and health

It is vital to explore how food and nutrition are understood and engaged with by Aboriginal peoples today. This provides opportunities to develop new ways of collaborating with Aboriginal peoples regarding food and nutrition policy, programs, and practice. This has the capability to reduce the impact of nutrition-related health issues such as diabetes on the lives of Aboriginal peoples and reduce health inequities, through the integration of Aboriginal nutritional knowledges and improved food systems and nutritional outcomes. It is often cited that given the large contribution of nutrition-related illnesses to the disproportionate health burden experienced by Aboriginal and Torres Strait Islander peoples, nutrition is an essential area for action (16, 25). Although we do not dispute that this is the case, we propose that *how* it is addressed needs to be transformed.

For example, for Aboriginal peoples, health is holistic, relational, and collective and has been described, as such:

Health does not just mean the physical well-being of the individual but refers to the social, emotional, spiritual and cultural well-being of the whole community. This is a whole of life view and includes the cyclical concept of life-death-life. (26)

Aboriginal and Torres Strait Islander health also has a greater reference to the whole of the life course, self-determination, community health, culture, dignity, justice, family, land, ties with the past, a vision for the future, hope, and stability (27, 28). Health has been described in the Dance of Life Paintings by Professor Helen Milroy as having 5 dimensions: physical, psychological, social, spiritual, and cultural (29, 30). In contrast, Western models of health have predominantly been based on the biomedical model which sees the body as a set of “parts” and “systems,” whereby a healthy body is one where all the “parts” and “systems” are working properly (31). In contrast to an Aboriginal and Torres Strait Islander view of health, this approach does not consider illness and disease within the context of the lives of people within communities (31) but rather views a person as compartmentalized and without their environments (27). These different understandings are important because they indicate fundamental differences in the ways in which health and well-being are understood. Because the dominant, mainstream approach to health care (including nutrition) in Australia is based on the biomedical model and/or a biopsychosocial model, there are severe limitations on the health system's ability to meet the full needs of Aboriginal and Torres Strait Islander peoples. Martin (32) writes about “the relational nature of our worlds and lives” including worldview, which inform and shape assumptions (p. 311). This worldview can be reset through research and experience (32, 33). Aboriginal and Torres Strait Islander understandings of health and healing provide an appropriate foundation from which to begin to reset the narrative in relation to nutrition.

One approach to value, include, and incorporate Aboriginal and Torres Strait Islander knowledges around food, nutrition, and health is through the Knowledge Interface. The Knowledge Interface was described by Maori Elder and Scholar, Sir Mason Durie, as the space where 2 different knowledge systems come together to create new knowledge that can be used to advance understanding in >1 system—in this case, Aboriginal and Torres Strait Islander understanding of food, nutrition, and health and non-Indigenous understandings (34).

Research at the Knowledge Interface is important for achieving action and outcomes because this is the space where complex issues can be considered in their entirety and meaningful collaboration and effective solutions achieved (35). Work at the Knowledge Interface is based on 4 principles: mutual respect, shared benefits, human dignity, and discovery (34). In Indigenous nutrition research, the Knowledge Interface can be used to position Aboriginal nutrition and food knowledge as relevant and as authoritative as Western nutrition and food knowledge in order to reset the narrative. It is a necessary theoretical and practical tool to avoid marginalizing Aboriginal and Torres Strait Islander perspectives and colonizing research processes which demean and disempower Aboriginal and Torres Strait Islander knowledge and peoples (36).

### Decolonization of Aboriginal and Torres Strait Islander Nutrition Research

Decolonizing theoretical perspectives acknowledges the ongoing effects of colonization on Aboriginal peoples, including negative health effects of historical and ongoing inequities (37, 38). Decolonization seeks to disrupt and challenge the ongoing colonial frameworks that marginalize and exclude Aboriginal knowledge, experience, and agency (at the individual and collective levels). Muller proposes 6 stages of decolonization including rediscovery and recovery, mourning, healing and forgiveness, dreaming, commitment, and action (39). It is vital to use decolonization as a theoretical perspective underpinning nutrition research with Indigenous peoples, and as a framework for action to transform structures and institutions (including the narrative around Aboriginal and Torres Strait Islander nutrition). If the decolonizing of the narrative around Aboriginal and Torres Strait Islander peoples’ nutrition is done in a way that privileges Aboriginal food systems and food knowledges, including the revitalization of such knowledge, then we propose there is real potential to maintain and strengthen cultural knowledge grounded in food systems, and there will be real and tangible benefits for families and communities around their nutritional welfare and outcomes. New research is required to identify what these benefits and outcomes will be. As the narrative will be based on diverse knowledges from Aboriginal and Torres Strait Islander peoples, it will be adaptable across diverse Aboriginal and Torres Strait Islander communities including urban, rural, and remote locations and different contexts.

Resetting the narrative around Australian Aboriginal and Torres Strait Islander nutrition research will require contribution from researchers, research funding bodies, and those working in the area of Aboriginal and Torres Strait Islander nutrition and health. Conducting, funding, and undertaking research with Aboriginal and Torres Strait Islander peoples that is strengths-based, inclusive of and framed by Aboriginal and Torres Strait Islander peoples and knowledges, and is decolonizing, will contribute to resetting the narrative around Aboriginal and Torres Strait Islander nutrition. This will enable more holistic understandings and solutions to address the multifarious phenomena affecting the nutrition of Aboriginal and Torres Strait Islander peoples and ultimately address the inequity in nutrition-related health conditions between Aboriginal and Torres Strait Islander peoples and non-Indigenous people in Australia.

## Notes

The authors reported no funding received for this study. AW, TM, and RW are funded by the National Health and Medical Research Council. ET is funded by 2 Flinders Foundation seeding grants.

Author disclosures: The authors report no conflicts of interest.
